# The association of leptin with dyslipidemia, arterial hypertension and obesity in Kyrgyz (Central Asian nation) population

**DOI:** 10.1186/1756-0500-7-411

**Published:** 2014-06-30

**Authors:** Erkin М Мirrakhimov, Alina S Kerimkulova, Оlga S Lunegova, Aibek E Mirrakhimov, Malik P Nabiev, Kseniya V Neronova, Asiyat A Bayramukova, Nazira T Alibaeva, Nurdin Satarov

**Affiliations:** 1 Kyrgyz State Medical Academy named by I.K. Akhunbaev, Akhunbaev street 92, Bishkek 720020, Kyrgyzstan; 2 National Centre of Cardiology and Internal medicine named by М. Mirrakhimov, Togolok Moldo 3, Bishkek 720040, Kyrgyzstan; 3 Kyrgyz-Russian (Slavic University), Kievskaya 44, Bishkek 720040, Kyrgyzstan

**Keywords:** Leptin, Abdominal obesity, Dyslipidemia, Arterial hypertension

## Abstract

**Background:**

Leptin, an adipocytokine produced by adipose tissue, along with the traditional cardiometabolic risk factors, contributes to the development of cardiovascular complications. At the same time, ethnic features of adipocytokines have been insufficiently investigated, especially among Asians, who have an increased risk of cardiovascular complications compared with Europeans. Aim of study was to investigate the relationship between leptin levels and age, gender, anthropometric parameters, lipid parameters, arterial hypertension (AH), and obesity in the adult population of ethnic Kyrgyz people living in Central Asia.

**Results:**

In total, 322 ethnic Kyrgyz (145 men, 177 women) aged ≥ 30 years were studied. Waist and hip circumference, body mass index, blood glucose, lipids, leptin, and homeostatic model assessment were measured. Patients in the upper quartile of leptin levels had high values of BMI, WC, systolic and diastolic blood pressure, glucose, and HOMA index compared with patients with lower leptin levels. The prevalence of metabolic syndrome and AH increased with higher levels of leptin. Leptin positively correlated with BMI, WC, triglycerides, and glucose concentrations in patients of both sexes. According to the multivariate logistic regression analysis, elevated leptin levels increased by 30 times the risk of obesity in men, regardless of the presence of type 2 diabetes, and 17.7 times in women.

**Conclusion:**

Leptin is associated with general and abdominal obesity, dyslipidemia, and insulin resistance in Kyrgyz patients.

## Background

High global prevalence of cardiometabolic diseases and the related mortality stimulated research focused on the risk factors, one of which is obesity [1-3]. It is well known that overweight and obese individuals have higher general as well as cardiac mortality [4]. Furthermore, obesity is strongly associated with the development of arterial hypertension (AH), insulin resistance (IR), type 2 diabetes mellitus (DM), atherogenic dyslipidemia, and other diseases [5].

The association between obesity and cardiometabolic risk factors may be mediated by the ability of adipocytes to synthesize biologically active substances with hormonal activity [6]. One of these hormones is leptin, which was identified in 1994 and has attracted the attention of obesity researchers [7]. Leptin is a 167 amino acid protein encoded by the obesity (OB) gene and is synthesized and secreted by adipocytes. In this case, serum leptin concentrations reflect the amount of energy reserves stored in adipose tissue [8]. In addition, leptin plays an important role in the regulation of feeding behavior and is closely associated with body mass index (BMI) and AH [9]. Leptin was shown to be associated with IR and other cardiometabolic risk factors in certain populations [10-12].

At the same time, obesity has been reported to have a different impact on metabolic risk factors and development of cardiovascular diseases in different ethnic groups [13]. It was also shown that among Asians, compared with Europeans, there is a higher incidence of coronary heart disease (CHD) [14]. In addition, cardiometabolic risk factors such as type 2 DM, IR, and abdominal obesity are often identified among Asians [15-17]. This raises the question of whether ethnicity influences the prevalence of cardiometabolic risk factors and cardiovascular disease, which has not been sufficiently studied.

Levels of leptin have never been studied in the ethnic Kyrgyz population. The purpose of this study was to investigate the relationship between leptin levels and age, gender, and anthropometric and lipid parameters in ethnic Kyrgyz adults.

## Methods

### Subjects

In 2008, we conducted a pilot cross-sectional study assessing the prevalence of cardiometabolic risk factors among residents of Kyrgyzstan. That study included 956 subjects who were later enrolled in the current investigation.

Exclusion criteria were age < 35 and > 70 years, conditions that could potentially alter leptin concentrations such as prolonged fasting, surgery within 1 month from study enrollment, advanced chronic diseases (such as chronic liver disease, chronic kidney disease, systemic autoimmune disease, congestive heart failure, thyroid disease, etc.), chronic use of glucocorticosteroids, use of lipid-lowering medications, patients with DM using insulin, pregnancy and lactation, chronic alcohol abuse, and people not of a Kyrgyz ethnic background. Thus, we included 322 ethnic Kyrgyz (145 men, 177 women), who signed informed consent to participate in the study, which included taking blood samples that were sent to France for analyses. The study protocol was approved by the local Ethical Committee of the National Center of Cardiology and Internal medicine, named after M.M. Mirrakhimov.

### Clinical examinations and laboratory analysis

All participants were examined by a cardiologist. The examination included taking the presenting complaints, medical history, physical examination with measurement of anthropometric parameters (height, weight, waist circumference [WC], hip circumference [HC], and blood pressure [BP]). BMI was calculated using the following formula: BMI = weight (kg)/height (m)^2^. Obesity was considered as a BMI ≥30 kg/m^2^ and overweight as a BMI of 25–29.9 kg/m^2^[18]. IR was calculated using the HOMA index values with the following formula: HOMA = serum insulin (μIU/ml) × plasma sugar (mmol/L)/22.5. A value of ≥2.77 was considered to be diagnostic for IR. Metabolic syndrome (MS) was defined according to modified ATP-III criteria [19]. All included patients filled out the Finnish Diabetes Association questionnaire to assess the risk of developing DM [20], which included information on vegetable consumption (every day or not every day) and physical activity (more or less than 30 minutes per day).

Blood samples were taken as previously described [21]. Laboratory tests included blood glucose (fasting), lipid profile (total cholesterol [TC], triglycerides [TG], high-density lipoprotein cholesterol [HDL-C], and low-density lipoprotein cholesterol [LDL-C]). All biochemical analyses were carried out in Dir adjoint du département Hommes, Natures, Muséede l'Homme (Paris, France). Leptin was determined using solid-phase enzyme immunoassay (EIA), and insulin by the enzyme-linked immunosorbent assay (ELISA)method (Bayer Corp). LDL-C was calculated by the Friedwald formula [22].

### Statistics

Statistical analysis was performed using SPSS 17.0 for Windows (SPSS, Inc., Chicago, IL). Variables with a normal distribution are presented as mean ± standard deviation and those with a nonparametric distribution as median (25^th^ and 75^th^ percentiles). The relationship between leptin and cardiometabolic risk factors was examined using Spearman rank order correlation. Analysis of variance (ANOVA) according to the Kruskal-Wallis method was used to compare the values (given the nonparametric distribution of variables). Post-hoc comparisons were performed by the Mann–Whitney test. Multivariate logistic regression analysis with stepwise inclusion of the variables was performed to identify the main factors influencing the development of obesity in the study group. Serum leptin concentrations were transformed into a natural logarithm to normalize the asymmetric distribution. A p-value < 0.05 was considered to be statistically significant.

## Results

### Clinical characteristics

Clinical characteristics of the patients enrolled in the study are presented in Table 1. The mean age was 51.7 ± 9.6 years. The prevalence of major cardiovascular risk factors (obesity, abdominal obesity, type 2 DM, smoking, and hyperlipidemia) was consistent with their prevalence in the sample from the primary study that was conducted in 2008 (please see the subjects section). However, the prevalence of AH was higher in the current sample (41.9%) compared with the initial group (36.2%). Nevertheless, systolic and diastolic BP were comparable in the two populations.

**Table 1:** Clinical characteristics of patients

	**n = 322**
Age, years	51,7 ± 9,6
Male, n (%)	145 (45)
Obesity, n (%)	94 (29,2)
BMI, kg/m^2^	27,4 ± 4,8
АО, n (%)	136 (42,2)
WC, cm	91,2 ± 11,5
HC, cm	101,4 ± 10,3
WC/HC	0,9 ± 0,08
AH, n (%)	135 (41,9)
SBP, mm Hg	135 ± 22
DBP, mm Hg	85 ± 12
Type 2 DM, n (%)	22 (6,8)
Fasting glucose, mmol/l	5,48 (5,1; 5,9)
TC, mmol/l	5,1 ± 1,1
HDL-C, mmol/l	1,1 ± 0,3
LDL-C, mmol/l	3,2 ± 0,9
TG, mmol/l	1,2 (0,9; 1,9)
МС, n (%)	107 (33,2)
Leptin, ng/ml	7,8 (4,0; 14,7)

For evaluation of the association between leptin levels and cardiometabolic risk factors, all participants in the study were stratified into four groups based on quartiles of leptin (< 2.2, 2.2–4.2, 4.3–6.34, > 6.34 ng/ml for males; < 8.05, 8.05–13.4, 13.5–19.09, > 19.09 ng/mL for females). As shown in Table 2, comparative analysis of the groups showed a significant difference in the age of the men: participants in the in the 4^th^ quartile were younger (p = 0.02). At the same time, it was observed that patients in the upper quartile of leptin had high values of BMI, WC, systolic BP and diastolic BP, glucose, and HOMA index compared with patients with lower leptin levels. The prevalence of MS and AH increased with higher levels of leptin. The above-described pattern was observed for the women as well, although the age was comparable in the groups of women. In addition, in contrast to the men, the groups of women showed no significant difference in TC, TG, LDL-C, as well as the occurrence of type 2 DM. The last was obviously due to the small number of patients with diabetes (Table 2).

**Table 2:** Characteristics of patients in association with leptin level*

**Men**	**1 quartile n = 39**	**2 quartile n = 35**	**3 quartile n = 35**	**4 quartile n = 36**	***p***
Age, years	52,5 ± 10,8	56,6 ± 9,6	55 ± 9,6	50,3 ± 8,5^†^	0,02
BMI kg/m^2^	22,9 ± 2,3	25,5 ± 2,5^##^	27,7 ± 2,2^##†^	29,8 ± 4,1^##††§^	0,0001
WC, cm	83,7 ± 8,1	92,7 ± 7,2^##^	98,9 ± 6,6^##†^	102,1 ± 11,6^##††^	0,0001
AH, n (%)	12 (30,8)	15 (42,9)^#^	17 (48,6)^#^	23 (63,9)^##^	0,0004
SBP, mm Hg	126 ± 18	139 ± 20^#^	139 ± 20^#^	148 ± 23^##^	0,0001
DBP, mm Hg	80 ± 12	84 ± 10	90 ± 12^#†^	95 ± 14^##††^	0,0001
TC, mmol/l	4,7 ± 0,9	5,3 ± 1,3	5,7 ± 1,1^#^	5,2 ± 1,03	0,004
TG, mmol/l	1,1 (0,8;1,5)	1,8 (1,2;2,4)^##^	1,5 (1,2;2,6)^##^	1,7 (1,2;2,4)^##^	0,000
HDL-C, mmol/l	1,13 ± 0,4	0,9 ± 0,3	1,0 ± 0,3	1,05 ± 0,3	0,4
LDL-C, mmol/l	3,04 ± 0,8	3,4 ± 1,3	3,8 ± 1,04^#^	3,3 ± 0,8	0,03
Type 2 DM, n (%)	0 (0)	4 (11,4)	6 (17,1)	4 (11,1)	0,08
Fasting glucose, mmol/l	5,3 (4,9;5,7)	5,5 (5,2;6,1)	5,6 (5,3;6,5)^#^	5,7 (5,2;6,5)^#^	0,046
НОМА index	0,9 (0,6;1,2)	1,5 (1,2;2,2)^##^	2,3 (1,6;3,1)^##†^	3,2 (2,2;4,5)^##††§^	0,0001
MS, %	15,4	25,7	37,1^#^	55,6^##†^	0,0005
**Women**	**n = 44**	**n = 45**	**n = 44**	**n = 44**	
Age, years	49,1 ± 11,1	49,8 ± 8,9	51,6 ± 8,9	50,6 ± 7,2	0,5
BMI kg/m^2^	23,6 ± 3,8	26,8 ± 2,8^##^	29,5 ± 3,6^##†^	33,3 ± 5,2^##††§§^	0,0001
WC, cm	78,7 ± 9,5	87,4 ± 8,0^##^	92,1 ± 8,7^##†^	97,5 ± 9,9^##††§^	0,0001
AH, n (%)	11 (25)	13 (29,5)	23 (51,1)^#^	20 (45,5)	0,01
SBP, mm Hg	127 ± 20	128 ± 21	140 ± 23^##†^	136 ± 19^†^	0,001
DBP, mm Hg	81 ± 10	82 ± 11	88 ± 12^##†^	86 ± 11	0,003
TC, mmol/l	4,8 ± 1,1	5,0 ± 0,9	5,1 ± 1,2	4,9 ± 0,9	0,7
TG, mmol/l	0,9 (0,7;1,3)	1,2 (0,9;1,4)	1,2 (0,9;1,8)^#^	1,1 (1,0;1,5)^#^	0,05
HDL-C, mmol/l	1,3 ± 0,4	1,2 ± 0,3	1,1 ± 0,3	1,2 ± 0,3	0,2
LDL-C, mmol/l	2,9 ± 0,9	3,2 ± 0,9	3,2 ± 1,0	3,2 ± 0,8	0,5
Type 2 DM, n (%)	3 (6,8)	3 (6,8)	1 (2,2)	1 (2,3)	0,5
Fasting glucose, mmol/l	5,2 (4,9;5,5)	5,5 (5,1;5,8)^#^	5,5 (5,2;6,3)^#^	5,6 (5,3;6,0)^##^	0,001
НОМА index	0,9 (0,8;1,4)	1,8 (1,4;2,6)^##^	2,3 (1,6;3,6)^##^	2,7 (2,1;4,0)^##††^	0,0001
MS, %	15,9	17,8	50^## †^	50^## †^	0,00005

*- quartile of leptine: men (< 2,2; 2,2-4,2; 4,3-6,34; > 6,34), women (< 8,05; 8,05-13,4; 13,5- 19,09; 19,09).

^#^- p < 0,05; ^##^ - p < 0,001 compared to the first quartile.

^†^- p < 0,05; ^††^- p < 0,001 compared to the second quartile.

^§^- p < 0,05; ^§§^ - p < 0,001 compared to the third quartile.

To study the association of leptin levels with BMI, patients were stratified into three groups: normal weight (BMI < 25 kg/m^2^); overweight (BMI ≥ 25–29.9 kg/m^2^); and obese (BMI ≥ 30 kg/m^2^). Comparative analysis revealed elevated leptin concentrations in men and women with increasing BMI (p < 0.001; Table 3).

**Table 3:** Leptin levels and BMI

**BMI kg/m**^**2**^	**n**	**Men (n = 145)**	**n**	**Women (n = 177)**
< 25	47	2,0 (1,4; 3,5)	45	6,3 (3,9; 8,5)
25-29,9	71	4,8 (3,2; 6,2)	65	13,3 (10,5; 17,2)
≥ 30	27	8,0 (5,7; 12,7)*	67	18,87 (15,7; 24,7)*

*- p < 0,001.

Next, we calculated Spearman correlation coefficients for leptin and anthropometric parameters, lipid profile, and blood sugar levels in men and women (Table 4). Leptin positively correlated with BMI, WC, TG, and glucose concentrations in patients of both sexes. At the same time, leptin was positively correlated with TC in men, and negatively correlated with HDL-C in women (Table 4).

**Table 4:** The Spearman correlation coefficient between leptin and independent factors

	**Men**		**Women**	
	***r***	**P**	***r***	**p**
Age	−0,063	0,45	0,127	0,09
BMI kg/m^2^	0,719	0,000	0,74	0,000
WC, cm	0,684	0,000	0,649	0,000
HC, cm	0,659	0,000	0,621	0,000
WC/HC	0,341	0,000	0,243	0,001
TC, mmol/l	0,214	0,01	0,09	0,235
TG, mmol/l	0,301	0,000	0,194	0,01
HDL-C, mmol/l	−0,079	0,3	−0,156	0,04
LDL-C, mmol/l	0,163	0,05	0,126	0,09
Fasting glucose, mmol/l	0,256	0,002	0,306	0,000

Multivariate logistic regression analysis was performed to assess the impact of various factors on the development of obesity and cardiovascular risk factors. Several regression models were analyzed, with the presence of obesity, AH, IR, DM as dependent variables and factors that may have an impact on their development (leptin, HDL-C, age, BMI, DM, AH, physical activity [more than 30 or less than 30 minutes a day] and vegetable consumption [every day or not every day]) as independent variables.

According to the analysis, elevated leptin levels increased by 30 times the risk of obesity in men, regardless of the presence of DM, and 17.7 times in women. In addition, physical activity of less than 30 minutes a day in women increased the risk of obesity by 3.2 times. A similar regression model constructed for AH showed that leptin levels increased by 2.1 times the risk of hypertension in men (p = 0.003; odds ratio [OR] 2.1; confidence interval [CI] 1.3–3.6) and in women (p = 0.01; OR 1.75; CI 1.2–3.8). However, an interconnected influence of leptin was lost after adjustment for BMI as an independent variable in the regression equation, probably due to obesity. Elevated levels of leptin also increased by 4.3 times the risk of IR in men and 6.9 times in women, regardless of BMI. The regression model for DM showed a trend for statistical significance (Table 5).

**Table 5:** Results of logistic regression models with stepwise inclusion of the variables: obesity, AH, IR и DM

	**ß**	**p**	**Expected ß**	**95% CI expected ß**
**Dependent - obesity**				
Men:				
Leptin	3,43	0,000	30,8	7,3 - 129,6
HDL-cholesterol	−3,48	0,007	0,031	0,002 - 0,39
DM	−2,097	0,07	0,123	0,03 - 1,21
Women:				
Leptin	2,87	0,000	17,71	6,64 – 47,2
HDL-cholesterol	−1,41	0,03	0,24	0,07 – 0,87
Low physical activity*	1,15	0,02	3,17	1,18 – 8,54
**Dependent – АH**				
Men:				
Age	0,053	0,008	1,054	1,01 – 1,096
BMI	0,217	0,000	1,24	1,12 – 1,38
Women:				
Low physical activity*	1,094	0,019	2,99	1,2 – 7,44
Age	0,,093	0,000	1,097	1,05 – 1,15
BMI	0,11	0,003	1,12	1,04 – 1,2
**Dependent– IR**				
Men:				
Leptin	1,45	0,000	4,25	2,14 – 8,43
HDL-cholesterol	−2,34	0,006	0,096	0,02 – 0,51
Low physical activity*	1,43	0,003	4,2	1,64 – 10,7
Women:				
Leptin	1,93	0,000	6,87	2,96 –15,96
HDL-cholesterol	- 3,18	0,000	0,042	0,01– 0,2
**Dependent – DM**				
Men:				
AH	1,41	0,041	4,09	1,06 –15,82
Women:				
AH	1,64	0,049	5,16	1,01 – 26,4

*Low physical activity – less than 30 min a day.

## Discussion

Unequal prevalence of cardiovascular disease in different ethnic groups is characterized by greater occurrence of cardiometabolic risk factors among Asians compared with Europeans [14]. Indeed, type 2 DM, IR, and abdominal obesity are frequently detected among Asians [15-17]. However, the available scientific publications are based mainly on research among South Asians [14-17]. Kyrgyz are people living in Central Asia, mainly in the highlands, with a different ancestral origin. Extrapolation of the results of the above-mentioned studies to the Kyrgyz ethnic group is not entirely correct, because there are significant differences between geographical living conditions and nutritional factors in the Kyrgyz compared with residents of South Asia (southern India, Pakistan, and Bangladesh).

In the present study, serum leptin concentrations were 3.1 times higher in women than in men. Similar sexual dimorphism was found in other studies (the difference ranged from 2.35 to 4.8 times) [23,24]. Gender differences in serum leptin concentrations are apparently due to several factors: the high content of fat in women [25-27]; structural differences in the hypothalamus [27]; and the influence of sex hormones. It is thought that androgens have a suppressive effect on leptin concentrations [27]. In addition, gender differences in leptin concentrations may also be affected by differences in body fat distribution. Central fat distribution is more common in men and peripheral fat distribution in women [28]. WC and HC are indicators of central and peripheral obesity, respectively [28]. Different studies revealed an association between leptin and regional adiposity. Leptin is more dependent on subcutaneous adipose tissue than on abdominal visceral tissue [29], since subcutaneous adipocytes secrete more leptin than omental fat tissue [30]. Studies have shown higher subcutaneous fat content in women than in men [31-33]. It is known that leptin levels reflect the amount of adipose tissue in the body, and hyperleptinemia is common in general and abdominal obesity. Similar results were obtained in the present study: serum leptin levels were significantly associated with abdominal obesity (AO), hypertriglyceridemia, and hyperglycemia. In addition, serum leptin levels positively correlated with BMI in patients of both sexes, although women had a higher BMI. The results of research on the relationship between serum leptin and lipids showed conflicting data. Some studies revealed no relationship between leptin and the parameters of a lipid profile [34,35]. Other studies showed a significant positive correlation between leptin and HDL-C [36,37] and TG [38]. In the present study, we found a positive correlation of leptin with TC in men and a weak negative correlation with HDL-C in women, probably due to the higher prevalence of hypercholesterolemia and decreased HDL-C in men and in women, respectively. At the same time, leptin levels were significantly correlated with TG in patients of both sexes. TG is stored in adipose tissue as the main form of energy among the biochemical markers, which explains its correlation with leptin [39-41].

In our study, we found an association of leptin with AH, which was confirmed in other studies such as the Olivetti Heart Study [42]. The prehypertensive effect of leptin was also shown in experimental animal research [43-46]. Apparently, such a relationship of leptin with AH is associated with the sympathetic nervous system, and its activation accompanies obesity in humans [47,48]. In our study, the logistic regression analysis revealed an association of leptinemia and AH, which disappeared after adjustment for BMI as an independent variable, indicating a relationship with obesity. Indeed, the physiological influence of leptin is shown in weight loss as reduced appetite and increased energy expenditure, as a result of the central stimulating effect on the activity of the sympathetic nervous system [49]. The kidneys are involved in the process. Studies have shown a clear association between circulating leptin levels and enhanced renal sympathetic stimulation [50], which was accompanied under experimental conditions by increased secretion of renin, and enhanced sodium and water reabsorption in the proximal tubule [51]. Despite that, the association between AH and obesity may be explained by the presence of IR and hyperinsulinemia [52]. In this study, we have shown a statistically significant association between blood leptin levels and IR in both men and women.

However, it is important to note that genetic factors influences the metabolism of leptin. For example, the 3’ UTR and Gln223Arg polymorphism of the leptin receptor were shown to play a role in the development of hyperleptinemia and AH [53,54]. Furthermore, impairment of β adrenergic receptor function, as well as G protein and calcium/calmodulin-dependent kinase IV play a role in the development of IR [21,55-57]. Unfortunately, we were unable to investigate the role of those genes in our study.

## Conclusion

Obviously, leptin as a biomarker for body fat reflects a yet unexplored activity of adipocytes and can provide important information regarding the risk of cardiovascular disease [58]. The results of our study show that leptin is associated with general and abdominal obesity, dyslipidemia, and IR. At the same time, further large-scale prospective studies are in great demand to better understand and closely investigate the physiological and pathological functions of leptin.

## Abbreviations

AH: Arterial hypertension; AO: Abdominal obesity; BMI: Body mass index; CHD: Coronary heart disease; DBP: Diastolic blood pressure; DM: Diabetes mellitus; HC: Hip circumference; HDL-C: High density cholesterol; IR: Insulin resistance; LDL-C: Low density cholesterol; MS: Metabolic syndrome; SBP: Systolic blood pressure; TC: Total cholesterol; TG: Triglycerides; WC: Waist circumference.

## Competing interests

The authors declare that they have no competing interests.

## Authors’ contributions

All authors contributed equally during the investigation process and article writing. All authors participated in manuscript discussion. All authors have read and approved the final manuscript. AEM performed English translation and revision of the manuscript.
